# TRP Channels Role in Pain Associated With Neurodegenerative Diseases

**DOI:** 10.3389/fnins.2020.00782

**Published:** 2020-08-04

**Authors:** Milena Duitama, Viviana Vargas-López, Zulma Casas, Sonia L. Albarracin, Jhon-Jairo Sutachan, Yolima P. Torres

**Affiliations:** Departamento de Nutrición y Bioquímica, Pontificia Universidad Javeriana, Bogotá, Colombia

**Keywords:** pain, Alzheimer’s disease, Parkinson’s disease, TRP channels, neurodegeneration

## Abstract

Transient receptor potential (TRP) are cation channels expressed in both non-excitable and excitable cells from diverse tissues, including heart, lung, and brain. The TRP channel family includes 28 isoforms activated by physical and chemical stimuli, such as temperature, pH, osmotic pressure, and noxious stimuli. Recently, it has been shown that TRP channels are also directly or indirectly activated by reactive oxygen species. Oxidative stress plays an essential role in neurodegenerative disorders, such as Alzheimer’s and Parkinson’s diseases, and TRP channels are involved in the progression of those diseases by mechanisms involving changes in the crosstalk between Ca^2+^ regulation, oxidative stress, and production of inflammatory mediators. TRP channels involved in nociception include members of the TRPV, TRPM, TRPA, and TRPC subfamilies that transduce physical and chemical noxious stimuli. It has also been reported that pain is a complex issue in patients with Alzheimer’s and Parkinson’s diseases, and adequate management of pain in those conditions is still in discussion. TRPV1 has a role in neuroinflammation, a critical mechanism involved in neurodegeneration. Therefore, some studies have considered TRPV1 as a target for both pain treatment and neurodegenerative disorders. Thus, this review aimed to describe the TRP-dependent mechanism that can mediate pain sensation in neurodegenerative diseases and the therapeutic approach available to palliate pain and neurodegenerative symptoms throughout the regulation of these channels.

Transient receptor potential (TRP) proteins constitute a group of non-selective cation channels (Gees et al., 2010) found in most cell membranes, except in the nuclear and mitochondrial membranes. TRPs are expressed in plasma membrane and help to modulate the driving force for the influx of Na^+^, K^+^, Ca^2+^, and Mg^2+^ ions, and trace metal ions (Nilius and Owsianik, 2011), while in specific organelles, such as the cilium and lysosomes, they regulate organelle and cellular activity (Moran, 2018). Numerous excitable and non-excitable tissues express TRPs, where they are involved in sensory signal transduction (nociception, taste, pressure, temperature, vision, and pheromone signaling), as well as homeostatic regulation (muscle contraction, vessel relaxation, and cell proliferation) (Gees et al., 2010). In the central nervous system (CNS), several TRP channels are expressed in both neurons and glia, fulfilling critical roles in neurogenesis, structural/functional plasticity, and cell homeostasis (Nilius, 2012; Vennekens et al., 2012; Katz et al., 2017).

It has been described that diverse ion channels expressed in the brain’s cells, including TRPs, are involved in the progression of neurodegenerative diseases such as Parkinson’s and Alzheimer’s. Also, several members of TRPs subfamilies are highly expressed in neurons and microglia mediating neuropathic pain (Haraguchi et al., 2012). TRP channels are part of cellular pathways related to the synthesis of many inflammatory mediators associated with neuroprotection/neurotoxicity, where they contribute to intracellular calcium regulation and signaling and painful stimuli transduction (Ji and Suter, 2007; Miyake et al., 2014; Lee and Kim, 2017). Therefore, TRP channels became of interest as promising targets for the treatment of both neurodegenerative diseases and pain.

In this review, we summarize the evidence of the role of TRP channels in the progression of neurodegenerative diseases such as Alzheimer’s and Parkinson’s diseases. Also, we discussed the possible involvement of TRP channels in pain associated with these neurodegenerative diseases and the use of TRP channels as possible pharmacological targets for pain treatment in patients with neurodegenerative diseases. A better understanding of the molecular mechanisms involved in neurodegeneration and pain is necessary to prevent and treat neurodegeneration and chronic pain.

## TRPs Structure and Expression

TRP multigene superfamily is formed by 28 members that encode integral membrane proteins that function as cation channels (Vennekens et al., 2012). TRP channels have some structural similarity, sharing as common a three-dimensional structure with six transmembrane segments (S1 through S6), two variable cytoplasmic domains (N- and C terminal), and small loop forming the channel pore between S5 and S6 segments (Catterall and Swanson, 2015). The distinguishing features between TRP channel subfamilies have been reported in the N- and C-terminal cytosolic domains, which contain residues and regulatory motifs unique for each family (Gaudet, 2008).

Unlike other cation-selective channel families, TRPs are classified by primary amino acid sequence rather than selectivity, ligand function, mechanisms of regulation, or sequence homology (Moran et al., 2004; Wu et al., 2010). TRP channels are divided into seven subfamilies: TRPC (Canonical), TRPV (Vanilloid), TRPA (Ankyrin), and TRPM (Melastatin), TRPP (Polycystic), and TRPML (Mucolipin). The seventh family, the no mechanoreceptor potential C channels (NOMPC or TRPN), is not found in mammals (Skryma et al., 2011). Alternatively, based on their sequence and topological features, TRP genes superfamilies are divided into Group 1 (TRPC, TRPV, TRPM, TRPA, and TRPN), and Group 2 (TRPP and TRPML). TRP subunits, in the same or different subfamilies, form functional homomeric or heteromeric ion channels with distinct biophysical and regulatory properties (Hellwig et al., 2005; Cheng, 2018). Heteromultimerization among mammalian TRP subunits have been observed for the TRPC, TRPV, TRPM, and TRPP families, displaying distinctive features (Hellwig et al., 2005; Cheng, 2018). For instance, formation of heteromeric complexes TRPC1/3, TRPC1/4, TRPC1/5, TRPC3/4, TRPC4/5 showed novel non-selective cationic channels with a voltage dependence or dynamic gating (Cheng et al., 2010; Kim et al., 2014; Woo et al., 2014). Also, TRPV1/3, TRPV5/6, TRPM6/TRPM7 or TRPML1/2 channels form heteromeric channels with intermediate conductance levels and gating kinetic properties (Cheng et al., 2007; Ma et al., 2011; Zhang et al., 2014; Goldenberg et al., 2015; Kim et al., 2016). Heteromerization within the mammalian TRP channel superfamily has also been observed. For instance, heteromeric TRPP2/TRPC1 and TRPP2/TRPV4 channels exhibit new receptor-operated property implicated in mechanosensation or thermosensitive roles (Du et al., 2014), and TRPC1/TRPC6/TRPV4 may mediate mechanical hyperalgesia and primary afferent nociceptor sensitization (Cheng, 2018).

The first TRP subfamily characterized was the canonical TRPC. The seven members of this subfamily are divided into four groups according to their sequence homology into Group I (TRPC1), group II (TRPC2), group III (TRPC3, TRPC6, TRPC7), and group IV (TRPC4 and TRPC5) (Nilius and Flockerzi, 2014). TRPC channels at the N-terminal domain show ankyrin repeats (3–4), a coiled-coil region, and a caveolin binding region. Meanwhile, the cytoplasmic C-terminal domain contains the TRP motif EWKFAR, a highly conserved proline-rich motif, and a region to interact with the IP_3_ receptor as well with calmodulin (calmodulin/IP_3_ receptor-binding region) (Putney et al., 2004). All TRPC are non-selective cation channels permeable to Ca^2+^ (Bon and Beech, 2013) linked to cellular processes such as cell division, differentiation, apoptosis, transduction of external stimuli, and refill of intracellular Ca^2+^ stores. In addition, they act amplifying receptor-activated Ca^2+^ signaling *via* interaction with second messengers (Numaga-Tomita et al., 2019). TRPC channels are widely distributed in cells of different tissues, including brain, heart, smooth muscle, liver, testis, ovaries, salivary glands (Beech et al., 2003), endothelium, kidneys (Freichel et al., 2005), and adrenal glands (Philipp et al., 2000). For instance, TRPC4/5 mRNA has been found in cortico-limbic brain regions, like the hippocampus and prefrontal cortex of adult rats (Fowler et al., 2007). TRPC channels are involved in diverse neuronal functions *via* receptor-mediated regulation by neurotrophic factors or neuropeptides, and cation influx through TRPCs control cellular functions and neuronal activity by regulating the membrane potential (Katz et al., 2017).

The TRPV subfamily is made up of six members, which are classified into four groups according to their homology: TRPV1/TRPV2, TRPV3, TRPV4, and TRPV5/TRPV6 (Smith et al., 2002; Xu et al., 2002; Nilius and Owsianik, 2011). TRPV channels were named after the discovery that its founding member (TRPV1) was activated by the vanilloid capsaicin, the compound responsible for a hot spicy taste (Szallasi and Blumberg, 1999). TRPV channels form homo- or hetero-tetramers, highly calcium selective, and mostly located on the plasma membrane. Each monomeric subunit typically contains three to five N-terminal ankyrin repeats and a TRP box at their C terminal. To this date, the most studied member of the TRP family is the TRPV1 receptor. TRPV1, TRPV2, TRPV3, and TRPV4 are moderately Ca^2+^ permeable, while TRPV5 and TRPV6 are highly selective Ca^2^^+^ channels and strictly regulated by [Ca^2^^+^]i (Gees et al., 2010). It is known that TRPV members have different gating properties, as studies using wild type and knockout mice models revealed that although TRPV2–6 channels share high sequence similarities with TRPV1, and they do not respond to temperature stimuli (Samanta et al., 2018). Furthermore, TRPV2 and TRPV4, unlike to other members of the family, are not sensitive to capsaicin (Caterina et al., 1999). TRPV1 channel is a homo-tetramer in which each monomer contains six ankyrin repeats in the N-terminal domain. The ion-conducting pore is formed by the transmembrane segments S5 and S6 and the pore-forming P loop and is similar to voltage-gated Na^+^ and K^+^ channels (Samanta et al., 2018). TRPV1 channels were first described in pain-sensitive neurons in dorsal root ganglia (DRG) and trigeminal ganglion neurons (Gees et al., 2010). Specifically, they are localized in peripheral small unmyelinated C- fibers, where they act as polymodal integrators of noxious stimuli in skin, muscles, joints, and internal organs (Samanta et al., 2018); also, TRPV2-4 channels are expressed in DRG neurons. TRPV3 is found in the brain, tongue, testis (Xu et al., 2002), skin, keratinocytes, and in cells surrounding hair follicles (Mandadi et al., 2009), while TRPV4 is expressed in non-neuronal cells like insulin-secreting β-cells, keratinocytes, smooth muscle cells, and different epithelial and bone cell types (Nilius et al., 2008).

The TRPA subfamily is constituted exclusively by the mammalian TRPA1 channel, first identified as an ankyrin-like transmembrane protein sharing similarities with other TRP channel subfamilies (Jaquemar et al., 1999). TRPA1 is a non-selective cation channel formed by homo- or hetero-tetramer subunits. The structure of human TRPA1 (hTRPA1) was determined by cryo-electron microscopy and shares a common structure with other TRP channels (Paulsen et al., 2015). TRPA1 has calcium-binding domains located in the C-terminal (Meents et al., 2019), 16 ankyrin repeat sequences in the N-terminal domain (Meents et al., 2017; Samanta et al., 2018), a putative selectivity filter located at the entrance of the pore, and a voltage sensor in the C-terminal (Meents et al., 2019). These domains allow TRPA1 channels to interact with other proteins, form molecular springs, and have better elasticity. This channel is expressed throughout the body, including the brain, heart, small intestine, lung, bladder, joints, and skeletal muscles (Kono et al., 2013). TRPA1 is highly expressed in DRG and trigeminal ganglia neurons (Takahashi et al., 2008) and acts as a mechanosensor in peripheral sensory pathways and the inner ear (Brierley et al., 2011).

TRPM channel subfamily consists of eight members grouped in four pairs: TRPM1 and TRPM3; TRPM2 and TRPM8; TRPM4 and TRPM5; and TRPM6 and TRPM7 (Fleig and Penner, 2004). All TRPM family members share common structural characteristics with other TRP channels (Fujiwara and Minor, 2008); however, they have a large cytosolic domain of between 732 and 1,611 amino acids for each subunit, which makes them the largest members of the TRP superfamily (Huang et al., 2020). Furthermore, unlike the TRPC, TRPV, and TRPA subfamilies, TRPM have a unique N-terminal (TRPM homology domain) without ankyrin repeats implicated in the channel assembly and trafficking (Kraft and Harteneck, 2005). Within subfamily members, the C-terminal section of TRPM channels is particularly variable, with TRPM2, TRPM6, and TRPM7, including active enzymatic domains (Samanta et al., 2018). TRPM2 has a nucleoside diphosphate pyrophosphatase domain (Chubanov et al., 2004) that specifically binds and hydrolyzes to ADP-ribose, while TRPM6 and TRPM7 contain α-kinase domains (Nadler et al., 2001; Drennan and Ryazanov, 2004). TRPMs are widely expressed in different tissues and organs; for instance, TRPM 2, 3, 4, 5, 6, and 7 are expressed in the CNS and periphery nervous system (PNS) (Mickle et al., 2015). Also, TRPM4, TRPM5, and TRPM8 are preferentially expressed in the prostate, while TRPM4, TRPM5, and TRPM6 are expressed in the intestine, and TRPM7 in heart, pituitary, bone, and adipose tissue (Bernardini et al., 2015). By contrast, TRPM1 is expressed by melanocytes and in malignant melanoma cells (Mickle et al., 2015).

**FIGURE 1:**
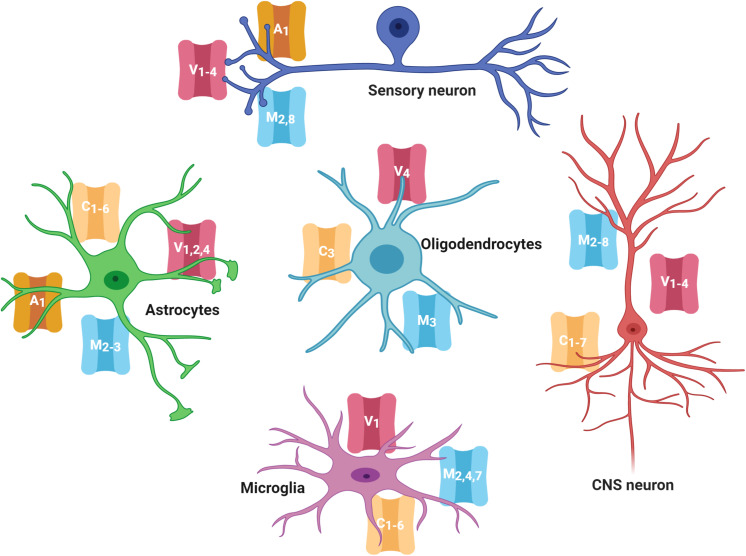
TRP channels expressed in the nervous system cells. Several members of the TRPC, TRPV, TRPM, and TRPA families are highly expressed in cells of the central and peripheral nervous system (neurons, astrocytes, oligodendrocytes, and microglia). TRP families are represented by capital letters as follow, C, TRPC; M, TRPM; V, TRPV; A, TRPA. Numbers indicates specific members of each family.

As mentioned before, several members of the TRPC, TRPV, TRPM, and TRPA families are expressed in neurons and glial cells in the CNS and PNS (Figure 1; Riccio et al., 2002; Moran et al., 2004; Abel and Zukin, 2008; Harteneck and Leuner, 2014; Zhang and Liao, 2015; Echeverry et al., 2016; Belrose and Jackson, 2018). Evidence has shown that TRPs in the CNS have critical roles in modulating growth cone guidance, synaptogenesis, synaptic plasticity, and in the development of several neurodegenerative diseases (Nilius, 2012; Vennekens et al., 2012; Katz et al., 2017). Notably, even when the role of TRPs in nociception in the PNS has been extensively described, their role in the CNS is almost unknown, and it has only recently gained attention.

## Activation Mechanisms of TRPs

TRP channels display a wide variety of activation mechanisms, which include physical stimuli, ligand binding, second messengers, and reactive oxygen and nitrogen species (Vennekens et al., 2012).

TRPC channels are modulated by a diverse group of second-messengers lipids that either regulate the channel activity or its insertion into the plasma membrane (Nilius and Szallasi, 2014). TRPCs activation mechanism converges various types of intracellular stimuli, including phospholipase C (PLC), protein kinase C (PKC) activity, diacylglycerol (DAG), intracellular calcium, and phosphatidylinositol 4,5-bisphosphate (PIP_2_) levels to modulate membrane potential and calcium input (Ramsey et al., 2006). Due to the flexible role of TRPC3 channel in calcium signaling and functional coupling with metabotropic receptors involving the PLC pathway in DRGs, as well as its regulation by pro-inflammatory molecules inducing channel sensitization (Séguéla et al., 2014), it has been of interest as a potential target for the management of chronic pain. Although most TRPCs are activated through PLC, which is a downstream effector of growth factors and neurotrophins, For instance, TRPC3, 6, and 7 are activated primarily by Gq/11 proteins, which are coupled downstream PLC-β; nevertheless, the Gα_i/o_ family are the dominant activators for TRPC4 and 5 (Nilius and Szallasi, 2014), which effectors of the PLC pathway are critical for the activation of TRPC channels remains a matter of debate, however, it is thought that specific TRPC channels may use different signaling effectors of this pathway (Putney, 2005). In this vein, it has been described that TRPC activation is dependent on recognition and lipid signals, and for instance, TRPC1, 2, 4, and 5 are activated by several DAGs (Lucas et al., 2003).

Besides lipid signaling, oxidative metabolism has a pivotal role in regulating TRPC channels activity (Kitajima et al., 2011) since they can be modulated by the production of reactive oxidative species (ROS) and reactive nitrogen species (RNS). TRPC channels can be considered redox-sensitive proteins that are targeted by ROS (Kim et al., 2013), and specifically, it has been reported that TRPC3 and TRPC4 are directly activated in response to oxidative stress (Aarts and Tymianski, 2005; Miller, 2006). It has been described that redox sensed by TRPC channels let the system indirectly to transduce lipid accumulation produced by the PIP_2_/DAG pathway (Malczyk et al., 2016), and the redox modifications of the lipid membrane environment that surrounds the channel (Poteser et al., 2006). For instance, TRPC3 activation by 1-oleoyl-2-acetyl-sn-glycerol (OAG) or mechanical stretch has shown to induce ROS production in rat neonatal cardiomyocytes (Kitajima et al., 2011). Additionally, it has been described in human embryonic kidney (HEK) cells that nitric oxide (NO) activates TRPC5 channels through mechanisms that require oxidation of extracellular cysteines in response to the NO donor S-nitroso-N-acetyl-DL-penicillamine (Yoshida et al., 2006). Also, intracellular oxidation regulates TRPC5 activation by glutathionylation, nitrosylation, and hydroxylation reactions, respectively, in Cys176 and Cys178 in contact with the intracellular redox environment, resulting in a sustained increase in [Ca^2+^]i and consequent cellular toxicity and neurodegeneration (Hong et al., 2015). In addition, oxidative metabolism also regulates the expression of TRPC channels (Song et al., 2011). Together ROS generation and Ca^2+^ signaling through TRPC channels modulate cellular processes that allow physiological and pathological responses in several organs (Malczyk et al., 2016) including kidney (Kim et al., 2013), brain (Hong et al., 2015), and the immune system (López-Requena et al., 2019). These factors have been associated in the pathogenesis of several chronic neurological disorders, including Alzheimer’s disease (AD) and Parkinson’s disease (PD), since ROS could activate cell death processes directly, through protein oxidation, lipids, and acting as second messengers in the cell death process (Gopalakrishna and Jaken, 2000; Nakamura and Lipton, 2009).

TRPV channels are activated by chemical ligands, such as capsaicin or cannabinoids, but also by noxious heat (>43°C), low pH (<6) (Caterina et al., 1997; Tominaga et al., 1998) and voltage changes inducing depolarization (Cao, 2020). TRPVs are also activated by lipid signaling (Cortright and Szallasi, 2004; Jung et al., 2004), and eicosanoids, signaling molecules produced by the enzymatic or non-enzymatic oxidation of arachidonic acid or other similar polyunsaturated fatty acids (Hwang et al., 2000). Specifically, TRPV1 activation can be achieved, regulated, and enhanced by several inflammatory molecules throughout metabolites downstream of G-protein coupled, such as PIP_2_ (Bhave et al., 2002), IP_3_ and DAG (Burgess et al., 1989), protein kinases such as PKA (Vlachová et al., 2003), PKC (Bhave et al., 2002; Varga et al., 2006), Ca^2+^/calmodulin-dependent protein kinase II (CaMKII) (Jin et al., 2004; Rosenbaum et al., 2004), and arachidonic acid metabolites like 12-HPETE (Shin et al., 2002).

It has been described that DRG neurons express TRPV1 to transduce and modulate pain stimuli in response to ligands and temperature (Caterina et al., 1997; Basbaum et al., 2009). Furthermore, it has also been shown that Bradykinin can regulate nociceptors such as TRPV1 activity, an inflammatory response mediator, that simultaneously stimulates the synthesis of PLC and its downstream targets (PIP_3_ and DAG), and arachidonic acid that further enhance cell excitation (Suh and Oh, 2005). During the inflammatory response, other pro-inflammatory mediators such as prostaglandins and sympathetic amines, sensitize nociceptors, including TRPV1, boosting pain sensation, or hyperalgesia (Zarpelon et al., 2013). A relationship between cytokines and oxidative stress has been found in hyperalgesia. For instance, NADPH oxidase leads to the production of superoxide anion by the TNF-α-induced NF-kB activation and consequentially causes overexpression of pro-inflammatory cytokines such as IL-1β (Possebon et al., 2014). Also, TNF-α and IL-1β activate cyclooxygenase-2 to produce prostanoids, which sensitize nociceptors, causing hyperalgesia (Verri et al., 2006).

TRPA1 channels have a wide range of natural and synthetic ligands (reactive electrophilic agonists) that induce channel gating by covalently bound to cysteine and lysine residues within the N-terminal and transmembrane domains, or promote the formation of C422–C622 disulfide bonds (Kimura, 2015). Also, polyunsaturated fatty acids (Viana, 2016), temperature (17–40°C) (Laursen et al., 2014; Moparthi et al., 2016) and changes in pH can activate TRPA1channels (Fujita et al., 2008; De La Roche et al., 2013; Zimova et al., 2018). De La Roche et al. (2013) reported activation of TRPA1, expressed in HEK 293T cells, with solutions above pH 5.4. However, it has been shown that in a Ca^2+^ dependent manner, pH between 7.4 and 8.5, also activates mouse TRPA1 channels heterologous expressed in HEK 293 cells (Fujita et al., 2008). Although the mechanism of how Ca^2+^ can modulate the sensitivity of the channel to more basic pH is still elusive, it has been shown that Ca^2+^ potentiates the activation and desensitization states of TRPA1 channels (Zimova et al., 2018). TRPA1 is a sensor for chemical irritants and a major contributor to chemo-nociception that is closely associated with TRPV1 channels, in terms of both expression and function (reviewed in Wang et al., 2019). Similarly to TRPV1, allogenic activators of TRPA1 channels are released from inflammatory environments or tissue injury sites to activate the channel (Chen and Hackos, 2015). For example, several lipid peroxidation products, oxidized lipids, and activators of the inflammasome, stimulate TRPA1 channels by an indirect mechanism involving H_2_O_2_ production (Trevisan et al., 2014). Additionally, endogenous lipidergic activators like nitrated fatty acids, produced by inflammatory processes, covalently bind to activate TRPA1 channels (Brewster et al., 2015).

TRPM activation mechanisms vary greatly among subfamily members, however, more than half of the members are sensitive to a wide range of temperatures, from cold to hot. For instance, TRPM4 and TRPM8 are activated by temperatures below 15 and 26°C, respectively (Talavera et al., 2005; Yao et al., 2011), while TRPM5 and TRPM2 are activated by temperatures above 35°C (Togashi et al., 2006). TRPM3 is the only member of this family that is activated by harmful heat, around 52°C in peripheral sensory neurons (Vriens et al., 2011). Some channels in this subfamily also respond to redox status, intracellular calcium, low temperatures, or ligands such as menthol. For instance, TRPM2 play a role in the transduction of oxidative stress stimuli (Oancea et al., 2011). In cortical neurons, TRPM2 channels are involved in the cytotoxic influx of Ca^2+^ that is induced by reactive oxygen species such as H_2_0_2_ (Kaneko et al., 2006). TRPM2 also activated by-products of nucleotides metabolisms like ADP-ribose (ADPR) and nicotinamide adenine dinucleotide (NAD) (Nadler et al., 2001; Hara et al., 2002; Kraft and Harteneck, 2005). It is not clear whether ROS directly or indirectly activates TRPM2 downstream of ADPR or NAD, however, recent evidence shows that oxidative stress triggers the production of ADPR mitochondrial that is released to the cytosol to activate TRPM2 (Perraud et al., 2005). It has been also described that H_2_O_2_ production after DNA damage, especially during certain phases of the cell cycle, induces an accumulation of 2′-deoxy-ATP mediated by an increase in NAD synthesis and a decrease in reserves of cellular ATP (Fliegert et al., 2017). The increased ratio of 2′-deoxy-ATP to ATP facilitates the synthesis of 2′-deoxy-NAD and subsequent hydrolysis to 2′-deoxy-ADPR. It is known that increasing amounts of cellular 2′-deoxy-ADPR mediates TRPM2 activation with similar potency but greater efficacy than ADPR, making it a TRPM2 super-antagonist (Fliegert et al., 2017). These findings are in congruence with the suggestions that TRPM2 activation under an oxidative environment could be related to pathological cell death in neurodegenerative diseases (Xie et al., 2010). TRPM4 and TRPM5 channels are activated by Ca^2+^, but they are not calcium-permeable (Oancea et al., 2011). The sensitivity of TRPM4 to intracellular Ca^2+^ is controlled by multiple signaling events, including ATP, PKC-dependent phosphorylation, calmodulin (CaM) binding, and membrane potential (Nilius et al., 2005). PIP_2_, Ca^2+^, and the voltage regulate the sensitivity of these channels, however, an increase in temperature in the range of 15 to 35°C further displaces the dependence of the voltage toward more negative potentials (Talavera et al., 2005). TRPM7 is also regulated by ROS and Ca^2+^ entry. Ca^2+^ has been considered as a relevant factor for the strong and lasting activation of TRPM7 in conditions of anoxia, oxidative stress, and metabolic imbalance, which could suggest mechanisms in which TRPM7 is involved and could induce even cell death (Aarts et al., 2003).

The PLC pathway, mediated by increased in intracellular calcium concentration, is an important mechanism involved in the modulation of some members of the TRPM family involved in depletion of PIP_2_ and the desensitization of TRPM4, TRPM5, TRPM7, and TRPM8 channels. Specifically, for TRPM4 it has been reported to cause a shift to the left of its voltage dependence and increase its sensitivity to Ca^2+^ 100 times (Owsianik et al., 2006). TRPM8 activation is inhibited by Gq-coupled receptors that mediate PLC activation, however, depletion of Ca^2+^ store activates chemical signaling through lysophospholipids (LPLs), enhancing TRPM8 activity (Vanden Abeele et al., 2006). Also, exogenous PIP_2_ (Liu and Qin, 2005), cold, or menthol (Rohács et al., 2005) activates TRPM8.

## TRP Channels and Neurodegenerative Diseases

Neuronal cell death rarely occurs in healthy brains, however, it can be triggered by internal/external factors in most neurodegenerative diseases (NDDs), where neurons initially lose their ability to maintain homeostasis due to changes in neuronal morphology, function, and viability (Dugger and Dickson, 2017; Chi et al., 2018). NDDs are categorized by their clinical features, anatomical structures affected, or molecular abnormalities (Kovacs, 2016). Although different in etiology, NDDs share common features, including mitochondria dysfunction, impaired energy metabolism, abnormal voltage-dependent anion channel activation, DNA damage, pro-inflammatory cytokines production, and disruption of cellular and axonal transport (Dugger and Dickson, 2017; Chi et al., 2018).

In the elderly, neurodegenerative diseases are a common and growing cause of mortality and morbidity, being AD and PD the most studied (Rahimi and Kovacs, 2014). AD is the most common form of dementia and makes up to 60–80% of all dementia cases worldwide, affecting an estimated 34 million people globally (Erkkinen et al., 2018). Meanwhile, PD affects 0.2 people per 100 of the population (independently of age), and almost 1–3% of the population older than 60 years (Tysnes and Storstein, 2017). Patients with AD or PD present learning and memory impairments, poor communication skills, irritability, symptoms of anxiety/depression, and progressive motor dysfunction (Batista and Pereira, 2016), and 40–85% of them suffer from painful conditions (Jost and Buhmann, 2019). Although the mechanisms that lead to these painful conditions are not fully understood, it is thought that neuropathological changes that occur in people with AD and PD dementia could alter pain perception (Van Kooten et al., 2016).

Calcium concentration level in neurons is exquisitely controlled to maintain cell homeostasis and to prevent neurodegeneration. The machinery that regulates intracellular Ca^2+^ levels is complex and includes several voltage-dependent plasma membrane calcium-conducting channels, glutamate receptors such as N-methyl-D-aspartate receptors and α-amino-3-hydroxy-5-methyl-4-isoxazolepropionic acid receptor, calcium release activated channels, and TRPCs. In addition, calcium flow from the endoplasmatic reticulum (ER) is highly regulated by Ryanodine receptors, Inositol trisphosphate receptor, calcium-dependent kinases, and phosphatases (Brini et al., 2014). Alterations in Ca^2^^+^ homeostasis have been related to the appearance and progression of several NDDs, including AD and PD (Marambaud et al., 2009; Nevzati et al., 2014). Indeed, it has been reported that exposure to either Aβ peptides (Li et al., 2009) or α-synuclein oligomers (Danzer et al., 2007) induces neuronal death by activating Ca^2+^dependent signaling pathways and metabolic derangements (Arundine and Tymianski, 2003), most likely by increasing mitochondrial Ca^2+^ levels and the release of proapoptotic factors (Orrenius et al., 2003; Beech, 2005

**FIGURE 2:**
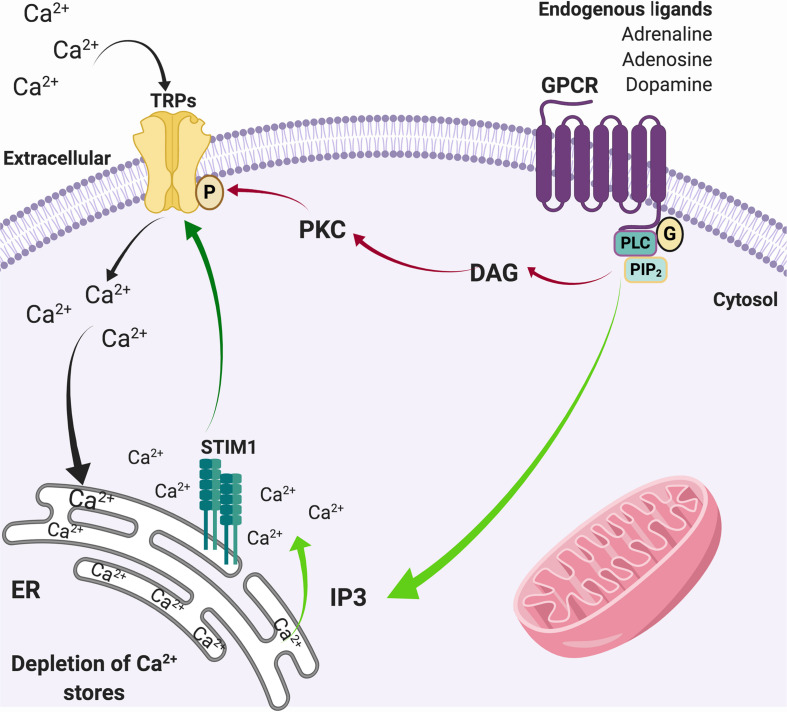
SOCE through TRP channels. Activation of G-protein coupled receptors activates the phospholipase C pathway that induces the hydrolysis of PIP_2_ to DAG (red arrows) that actives PKC, which in turn phosphorylates TRP channels. In parallel, the generation of IP_3_ (green arrows) promotes the release of Ca^2+^ from the ER. The depletion of intracellular Ca^2+^ stores from the ER is sensed by STIM1, which also activates Ca^2+^ channels in the plasma membrane such as TRPs (dark green arrow), allowing the entry of Ca^2+^ from the extracellular medium to the cytosol (black arrows) to refill de ER deposits.

**FIGURE 3:**
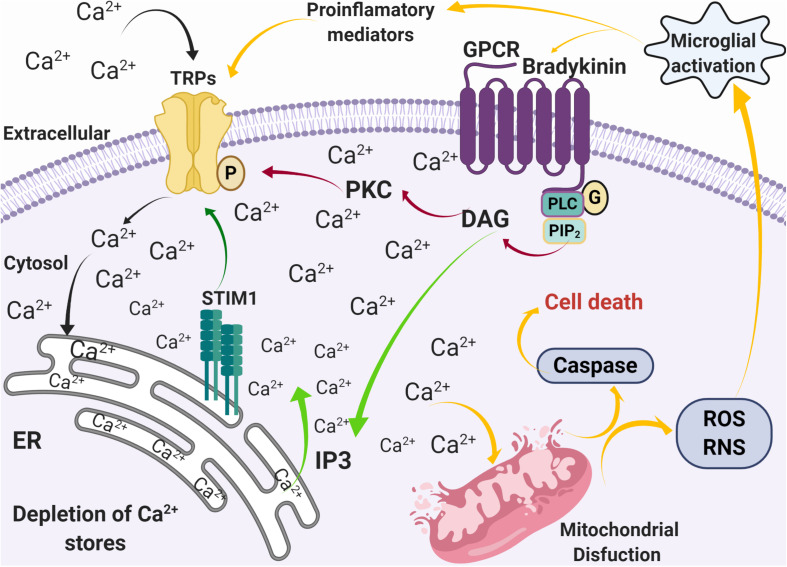
Alterations in calcium homeostasis mediated by SOCE during inflammation and oxidative stress. Activation of G-coupled receptors by pro-inflammatory mediators, such as bradykinin, induces the release of Ca^2+^ from the ER stores through the PLC pathway (green arrows), followed by an influx of Ca^2+^ through Ca^2+^ permeable channels such as TRPs (black arrows). The increase [Ca^2+^]i then induces mitochondrial dysfunction that leads to caspase activation, ROS and RNS production, microglia activation, and production of pro-inflammatory mediators (yellow arrows).

In physiological conditions, activation of G-coupled receptors at the plasma membrane induces the release of Ca^2+^ from the ER, which in turn stimuli the influx of extracellular Ca^2+^ through a diversity of plasma membrane channels. This process is known as store-operated Ca^2+^ entry (SOCE) (Putney, 1986). SOCE calcium fluxes are mediated by calcium selective ion channels ORAI (calcium release-activated calcium channel proteins) (Kraft, 2015) that allow the calcium release-activated calcium current (I_CRAC_) and store-operated calcium current (I_SOC_) mediated by relatively selective Ca^2+^ to non-selective cation channels, such as TRPC1/4/5 (Parekh and Putney, 2005; Yuan et al., 2007). It has been suggested that Orai binds to TRPC1 and the stromal interaction molecule 1 (STIM1) during SOCE activation, enhancing calcium currents (Liao et al., 2008; Zhang et al., 2016). In this regard, TRPCs play a role in [Ca^2+^]i regulation by modulating SOCE (Minke and Cook, 2002), which joint to other TRPs, such as TRPC3, TRPC4, TRPM2, and TRPM7, respond to oxidative stress (Selvaraj et al., 2012), and may contribute to neurodegeneration (Figure 2). Given the expression of TRP channels in brain regions damaged during the development of PD and AD, and their role in Ca^2+^ homeostasis and ROS/RNS sense, they are now considered key players in neuronal degeneration and potentially on altered pain perception (Figure 3) (Bernd and Appendino, 2007; Nilius and Flockerzi, 2014; Rojo et al., 2014).

## TRPs in Parkinson’s Disease

PD is characterized by a marked loss of dopaminergic neurons (DNs) in substantia nigra (SN) (Cacabelos, 2017). Although the mechanism by which these neurons degenerate is not well known, mitochondrial dysfunction, oxidative stress, inflammation, altered calcium homeostasis, NO synthesis, protein aggregation, excitotoxicity, and glutathione (GSH) depletion (Mandel et al., 2003), and activation of microglia-mediated by glucocorticoid receptors (GR) (Maatouk et al., 2018), are related to degeneration of DNs (Channels, 2017). Considering that oxidative stress and changes in Ca^2+^ homeostasis are involved in PD, it has been suggested that TRP channels could mediate some of the mechanisms that lead to the development of the disease.

Kim et al. (2005) showed that capsaicin, a TRPV1 agonist, elicits cell death of mesencephalic DNs. Additionally, it has been reported that TRPV1 activation triggers Ca^2+^-dependent cell death (Kim et al., 2006) and NADPH-oxidase-mediated production of ROS in microglia (Shirakawa and Kaneko, 2018), suggesting that a similar mechanism could operate in death of DNs in PD. For instance, TRPV1 antagonists such as capsazepine and iodo-resiniferatoxin inhibit DNs death *in vivo* and *in vitro* (Kim et al., 2005). Mechanistically, it is thought that TRPV1 activation induces an increase in [Ca^2+^]i that impairs mitochondrial function, induces cytochrome release, and caspase-3 cleavage. Consequently, activation of TRPV1 channels contributes to dopaminergic neuron damage *via* Ca^2+^ signaling and mitochondrial disruption (Kim et al., 2005). Although the nature of the endogenous ligands that induce the activation of TRPV1 in PD has not been elucidated, these channels are endogenously activated by anandamide, an endocannabinoid, which is increased in untreated PD patients (Pisani et al., 2010).

In contrast to the toxic role of TRPV1 activation on DNs, TRPC1 has been suggested as a protector and critical mediator of DNs survival (Sun et al., 2012). DNs are characterized by a pacemaker activity that is thought to be dependent on the activation of the Ca^2+^ channel Ca_v_1.3 and Na^+^ channels. Interestingly, Cav1.3-mediated cell death is prevented by translocation of stromal interacting molecule-1 (STIM1) induced by Ca^2+^depletion of ER, allowing it to interact with and activate calcium permeable channels like TRPC1 to refill the ER Ca^2+^ store (Soboloff et al., 2012; Kraft, 2015). That process protects DNs against the Cav1.3-mediated cell death. Neurotoxins that mimic PD symptoms, such as 1-methyl-4-phenyl-1, 2, 3, 6-tetrahydropyridine (MPTP), increase the activity of the Cav1.3 channel by downregulating the expression of TRPC1, which lead to a decrease in SOCE and the release of Ca^2+^ from ER to the cytosol in DNs and mesenchymal stem cells (Sun et al., 2018). It has been described that MPP^+^ (1-methyl-4-phenylpyridinium), a toxic metabolite product of enzymatic activity of MAO-B on MPTP, kills DNs in SN (Choi et al., 2015). In this vein, it has been shown that Cav1.3 silencing or TRPC1 overexpression decreases caspase 3 and inhibits MPP^+^-induced cell death. Therefore, TRPC1 expression facilitates STIM1-Cav1.3 interaction, and it is essential for the survival of DNs in PD (Sun et al., 2017). Moreover, Chen et al. (2013) found that the downregulation of Homer 1 protein inhibited the generation of ROS induced by MPP^+^ in DNs, without affecting the activity of endogenous antioxidant enzymes; this inhibition was further potentiated by BAPTA-AM. Exposure of DNs to MPP^+^ induces a rapid increase in cytosolic Ca^2+^ concentrations after its release from the ER, an effect that was prevented in DNs with low Homer1 expression (Chen et al., 2013).

Beyond its role in DNs-induced cell death, MPP^+^ can directly activate microglia and promote the production of several pro-inflammatory mediators and iNOS (Kim et al., 2018; Lee et al., 2019). Once microglia are activated, the release of pro-inflammatory microglial cytokines and chemokines induce the death of dopaminergic (DA) neurons, evidencing the vulnerability of these neurons to glia-mediated neurotoxicity. In the MPTP model, M1 microglia have been associated with dopamine neurodegeneration by the induction of microglial NOS and NADPH oxidase (NOX) (Appel et al., 2015). Mizoguchi et al. found that brain derived neurotrophic factor (BDNF) induces a sustained elevation of [Ca^2+^]i through the overregulation of TRPC3, which is also crucial for the suppression of NO induced by BDNF-activated microglia. This signaling pathway has been linked to the inflammatory response that mediates DA death in PD (Mizoguchi et al., 2014).

Furthermore, Parkinsonian disorders are often associated with changes in the frequency and firing mode of GABAergic neurons (Zhou et al., 2008). In SN and Globus Pallidus internus, GABAergic neurons project and regulate the firing pattern of thalamic nuclei, superior colliculus, and brainstem motor nuclei, regulating the smoothness and coordination of movements (Zhou et al., 2008). TRPC3 channels selectively expressed in the SN GABA projection neurons regulate the firing pattern of these neurons. The expression of TRPC3 in SN maintains a constant influx of Na^+^ that generates a tonic depolarized potential that contributes to the high frequency and regularity pattern of firing of these neurons (Zhou et al., 2008). However, it has been described that ROS-induced increased TRPC3 activity could lead to a more depolarized potential in GABAergic projecting neurons, contributing to the unbalance of disinhibition and inhibition cycles observed in PD (Zhou et al., 2008).

TRPM7, a Zn^2+^, Ca^2+^, and Mg^2+^ permeable channel, has been associated with NDDs given its regulation by intracellular Mg^2+^ levels and ROS (Nadler et al., 2001; Sun et al., 2015). PD animal models have shown that Mg^2+^ deficits increase the vulnerability of DNs to MPTP neurotoxicity (Muroyama et al., 2009). Furthermore, Mg^2+^supplementation inhibits the toxicity of (methyl-4-phenylpyridium ion) by decreasing the death of DNs and maintaining the length of their neurites. These results are in agreement with the observation that TRPM7 is significantly decreased in the SN of PD patients and that long-term Mg^2+^ deficiencies significantly decrease the number of DNs in SN (Oyanagi et al., 2006). These results suggest that DNs utilize TRPM7 channels to regulate Mg^2+^ levels, and that loss of TRPM7 channel function may be involved in the development of PD (Landman et al., 2006).

It has been reported that PD patients have significantly elevated cortisol levels compared to control subjects of the same age (Bellomo et al., 1991; Ros-Bernal et al., 2011). Interestingly, expression of TRPM6 and TRPM7 can be regulated by glucocorticoids (GCs) in a tissue-dependent manner (Cuffe et al., 2015). In the brain, GC signaling is mediated by GRs well as by mineralocorticoid receptors expressed in neurons and glia (Sierra et al., 2008). A large number of studies indicate that activation of GRs by GC promotes inflammatory response (Bhattacharyya et al., 2010), particularly in microglia (Maatouk et al., 2018). For instance, inflammation caused by a low dose of Lipopolysaccharides (LPS) directly administrated in substantia nigra causes a specific loss of dopaminergic neurons (Castaño et al., 2002). Interestingly, pre-treatment with a low dose of dexamethasone (DXM, 1 mg/Kg) diminished nigrostriatal dopaminergic neurons damage in mice treated with 1-methyl-4-phenyl-1,2,3,6-tetrahydropiridine (MPTP, 40 mg/Kg), while a high dose of DXM (10 mg/Kg) further aggravate loss of dopaminergic neurons (Kurkowska-Jastrzȩbska et al., 2004). However, physiological levels of GC and functional response of GRs are necessary to prevent neurodegeneration; indeed, it has been reported that in the absence of GR, microglia-induced dopaminergic neuronal loss (Barcia et al., 2011).

## TRPs in Alzheimer Disease

Altered Ca^2+^ homeostasis has been considered one critical factor regulating neuronal death in AD (Small, 2009). For instance, mutations in presenilins, catalytic subunits of the gamma-secretase, have been linked to Ca^2+^ signaling dysregulation, proteolytic processing of amyloid precursor protein (APP), and thereby increasing production of Aβ peptide (Guo et al., 1997, 1999; Schneider et al., 2001; Banerjee and Hasan, 2005). Aggregation of the Aβ peptide may induce the release of Ca^2+^ stored in the ER, resulting in an overload of cytosolic Ca^2+^. In response to the rise in [Ca^2+^]i, endogenous levels of GSH are reduced, leading to a ROS accumulation within cells (Ferreiro et al., 2008). In addition, the deposition of Aβ also induces microglial activation (Seabrook et al., 2006) and the release of pro-inflammatory cytokines, initiating pro-inflammatory signaling pathways that subsequently contributes to neuronal damage and death (Wang et al., 2015). Pro-inflammatory cytokines also sensitized TRPV1 channels expressed in a variety of cells, such as microglia, astrocytes, pericytes, and neurons (Tóth et al., 2005), suggesting that these channels contribute to AD-related neuroinflammatory processes. Inhibition of TRPV1 dependent generation of ROS significantly diminishes the detrimental effect of activated microglia and the inflammatory response elicited by astrocytes upon stimulation with the Aβ peptide (Harada and Okajima, 2006; Benito et al., 2012). However, capsaicin activation of TRPV1 protects the hippocampus function by rescuing the effect of Aβ peptide on the hippocampal gamma oscillations (Balleza-Tapia et al., 2018). These differences compared to the response of TRPV1 after activation by capsaicin, could be accounted for by experimental conditions, likely related to β-amyloid concentrations used in both studies (Balleza-Tapia et al., 2018). Purely fibrillary beta-amyloid preparations have been reported to be more toxic in some experimental models (Kurudenkandy et al., 2014; Cohen et al., 2015), and this possibly induces pathological activation of inflammatory mechanisms, mediated by TRPV1 in primary astrocyte culture (Devesa et al., 2011; Tsuji and Aono, 2012).

In the brain, TRPA1 channels play an essential role in their development and function of non-neuronal cells, such as astrocytes (Shigetomi et al., 2012, 2013). Although AD is a complex disease in which several mechanisms may act, recent studies have evaluated the role of Ca^2+^ related signaling pathways in the etiology and development of the disease (Yamamoto et al., 2007; Takada et al., 2013). Lee et al. (2016) demonstrated *in vitro* that Aβ triggers a TRPA1-dependent Ca^2+^ influx and astrocytic activation. Additionally, ablation of TRPA1 in APP/PS1 transgenic mice slowed the progression of AD and improved learning and memory performance, and reduced Aβ plaques and cytokines (Lee et al., 2016). These results have been further supported by TRPA1 expression in HEK cells, where Aβ is also capable of inducing TRPA1 dependent Ca^2+^ signaling, that activate transcription factors such as NF-κB and NFAT and promote expression of pro-inflammatory cytokines (Lee et al., 2016).

Interestingly, loss-of-function or pharmacological inhibition of TRPM2 channels prevents microglial activation and TNF-α production induced by a wide range of Aβ42 concentrations (10–300 nM), proving a novel role of TRPM2 in microglial activation triggered by Aβ42 peptides (Alawieyah et al., 2018). Likewise, Ostapchenko et al. (2015) demonstrated that TRPM2 ablation in AD models decreases microglial activation, improves the expression of synaptic markers and reduces the deficits in memory observed in aging animals (Ostapchenko et al., 2015). Furthermore, it has been shown that TRPM2 endogenous expression in rat striatum neurons and activation by Aβ and oxidative stress is enough to drive cell death, suggesting that TRPM2 is an active transducer of ROS signaling that may contribute to neuronal death in AD (Fonfria et al., 2005). At a cellular level, ROS levels are regulated by a complex mechanism that involves antioxidant enzymes and small-molecule antioxidants such as GSH (Geon et al., 2015). GSH levels tend to be lower with age and have been considered as markers of cognitive impairment severity (McCaddon et al., 2003). Interestingly, in neuronal cultures that recapitulate aging, GSH supplementation significantly decreases TRPM2 expression and activity (Sita et al., 2018). Therefore, downregulation of the antioxidant defense plus the Aβ-induced production of ROS and cytokines in AD can lead to the activation of several TRP channels that can increase [Ca^2+^]i, leading to excitotoxicity and apoptosis (Park et al., 2014**).**

Although some advances have made in understanding the role of TRP channels in neurodegenerative diseases, we are still far from having an integrated comprehension of the role of these channels in the etiology and development of these diseases. For instance, more studies are needed to unveil how all these channels work together either to degenerate or protect neurons in PD and AD.

## TRPs Involvement in Pain, Alzheimer’s, and Parkinson’s Diseases

During the past decade it has been an increasing awareness of pain and pain management as important issues to address in the elderly (Ali et al., 2018) and patients with neurodegenerative diseases (Cravello et al., 2019). Pain symptoms in NDD patients include sleep disorders, musculoskeletal problems, reduced mobility, falls, malnutrition, cognitive impairment, increased drugs use, diminished social behavior, anhedonia, and depression (Cravello et al., 2019). Prevalence of painful symptoms in patients with AD range from 38 to 75%, and from 40 to 86% in PD (Batista and Pereira, 2016; Van Kooten et al., 2016; de Tommaso et al., 2017; Cravello et al., 2019).

Even when PD was previously considered as a purely motor disorder, now it is known that non-motor symptoms, including pain, occur throughout the course of the disease and significantly affect the quality of life (Jost and Buhmann, 2019). Some nociceptive pain associated with PD is a secondary consequence of the motor impairment (abnormal muscular tone, spasms, rigidity, reduced active mobility, osteoarticular problems, and local inflammation), however, as many as 43% of Parkinson patients exhibits characteristics typical of neuropathic dysfunction (burning, tingling, formicating, decreased nocifensive flexion reflex, and lowered cold threshold) (Reichling and Levine, 2011; Skogar and Lokk, 2016; de Tommaso et al., 2017). Neuropathic pain has been recently studied in a model of nigro-estriatal pathway lesion, which induces allodynia and hyperalgesia in rats (Romero-Sánchez et al., 2019).

Similarly, it has been described that pain is more prevalent in AD patients, and that intensity of pain is also positively correlated with dementia severity (Cao et al., 2019). Typical cognitive impairment observed in AD also affect the assessment of a painful experience and the ability to describe it (Cravello et al., 2019). It has been reported that neural circuits mediating pain perception and its behavioral expression may be hyperactive or underactive in AD: Specifically, altered pain response seems to depend on the extension of the brain tissue damage, stage of the disease, and type of pain (acute stimuli or chronic medical conditions) (Monroe et al., 2012).

Recently, neuropathological changes occurring during the progress of dementias are being considered as possible causes of pain perception alterations (Cravello et al., 2019), and it has been suggested that primary neuropathic pain is not a simple consequence of nervous system deterioration but instead the result of the very same cellular processes that underlie neurodegenerative diseases (Reichling and Levine, 2011; Cravello et al., 2019). The neuropathological changes that occur in AD affect structures comprised in CNS processing affective-motivational (hippocampus, entorhinal cortex, cingulate gyrus, hippocampus, amygdala), cognitive-evaluative (prefrontal cortices), and sensory-discriminative (somatosensory cortex) aspects of pain (Monroe et al., 2012; Achterberg et al., 2013; Dugger and Dickson, 2017). Similarly, in PD, insufficient input from dopaminergic neurons to basal ganglia and motor and prefrontal cortices results in enhanced inhibitory inputs, which leads not only to body movement-related symptoms but also cognitive and emotional symptoms associated to altered pain perception (Chi et al., 2018).

**FIGURE 4:**
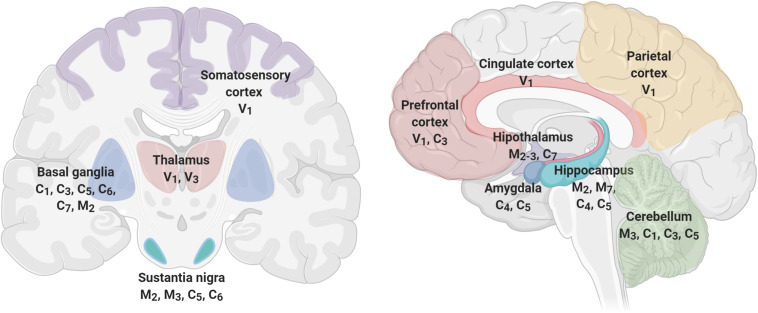
TRP channels are expressed in brain structures involved in pain perception. Pain processing includes cortical (prefrontal, parietal, somatosensory, and cingulate), limbic (amygdala, hippocampus, thalamus, hypothalamus), and movement-related structures (Basal Ganglia, Substantia Nigra, and Cerebellum) that express several members of the TRP channels. TRP families are represented by capital letters as follow, C, TRPC; M, TRPM; V, TRPV; A, TRPA. Numbers indicates specific members of each family.

Figure 4 shows brain’s structures involved in pain perception, which include the prefrontal cortex, hippocampus, amygdala, entorhinal cortex, anterior cingulate cortex, basal ganglia, thalamus, insula, and sensory cortex (Fenton et al., 2015; Mano and Seymour, 2015; Cao et al., 2019), and TRP channels expressed in each one of these structures (Kauer and Gibson, 2009; Harteneck and Leuner, 2014; Nilius and Szallasi, 2015; Frias and Merighi, 2016; Katz et al., 2017). As described before, TRP channels have an unique role in nociceptive, neuropathic, and inflammatory pain as diverse members of their families are involved in pain pathways (Hung and Tan, 2018). For instance, members of TRPA, TRPV, and TRPM subfamilies have high expression levels in neurons mediating neuropathic pain (Naziroğlu, 2012). Interestingly, members of the TRPC and TRPM families are expressed in SN, basal ganglia, and hippocampus, brain structures that exhibit significant loss of neurons at the initial stages of the development of AD or PD, respectively. The specific role of TRPs on NDD-related pain symptoms have not been thoroughly studied. However, several lines of evidence indicate a relationship between pain, neurodegeneration and TRPs, particularly related to inflammation.

At the molecular level, it has been proposed that pain-related to NDDs is associated, not only to loss of selected neuronal population but to microglial activation, that response to noxious stimuli realizing inflammatory mediators such as pro-inflammatory cytokines, interleukins, and tumor necrosis factor alpha (TNFα) (Carniglia et al., 2017). Notably, chronic pain (inflammatory or neuropathic pain) related to neurodegeneration is also accompanied by neuroimmune activation and an escalated response that impairs homeostatic balance since anti-inflammatory mediators are not released, inducing further tissue damage, neuroinflammation, and neurodegeneration (Carniglia et al., 2017; Salter and Stevens, 2017; Inoue and Tsuda, 2018). The role of glial cells in the initiation, sensitization, and maintenance of chronic pain has been studied during the past two decades (Cravello et al., 2019), and it has been found that neuromodulators produced by microglia can rapidly alter synaptic plasticity, a driving force for the pathogenesis of pain after tissue or nerve injury (Chen et al., 2018).

During inflammatory pain, inflammatory molecules can change the TRP threshold activation, inducing mechanical allodynia, thermal hyperalgesia, and spontaneous pain. TRPV1, TRPA1, and TRPM2 channels have been intensely studied in pain sensation because they participate in the cellular signaling mechanism through which injury produces pain hypersensitivity. These channels can be activated by thermal stimuli and endogenous molecules derived from the inflammation process (Ma and Quirion, 2007; Hung and Tan, 2018). After an injury, inflammatory molecules such as eicosanoids, neuropeptides, and cytokines decrease the thresholds of sensory neurons, inducing sensitization in TRPV1 (Julius, 2013). As TRPA1 is highly regulated by oxidative stress and is targeted by different reactive species, so that they are activated during inflammatory progression, where ROS produced after tissue injury induces superoxidation of membrane phospholipids and activation of the channel (Julius, 2013; Mori et al., 2016; De Logu et al., 2017; Hung and Tan, 2018). The role of TRPM2 in pain generation is through their activation by reactive nitrogen species (Kaneko et al., 2006). Similar to TRPV1, TRPM2 suffers sensitization by H_2_O_2_ that lowered the temperature of activation (Kashio et al., 2012). Interestingly, it has been described that chronic pain is a risk factor to develop memory impairment, dementia, and other neuropsychiatric conditions (Moriarty and Finn, 2014; Whitlock et al., 2017).

TRP channels expressed in sensory neurons have an essential function in pain and inflammation transduction (Fernandes et al., 2012; Smani et al., 2015). Similarly, it has been reported that microglial TRP channels have a significant role in pain modulation as well as in AD and PD (Cravello et al., 2019) by regulating the levels of ROS, pro-inflammatory cytokines, and the homeostasis of Ca^2+^. All these processes are connected with microglial activation, which is a cellular process proposed as a central player in both pain and neurodegenerative diseases (Miyake et al., 2014; Echeverry et al., 2016). For example, during inflammation, an upregulation of TRPM2 channels in microglia leads to an exacerbated inflammatory response mediated by ROS. This mechanism has been proposed as one of the primary inductors of inflammation and neuropathic pain (Haraguchi et al., 2012). However, It has also been shown that TRPV1 channels protect mesencephalic DA neurons by inhibiting microglia-originated oxidative stress, suggesting that TRPV1 channels may be novel targets for regulating the oxidative stress-mediated neurodegeneration observed in PD (Park et al., 2012).

TRP-dependent microglial activation involves the influx of Ca^2+^ and the activation of Ca^2+^ -mediated signaling pathways that induce the synthesis of pro-inflammatory molecules, including interleukins (IL-1β and IL-12), chemokines, prostaglandins (PGs), TNF-α, ROS, and NO. These molecules promote an exacerbated inflammatory response by the recruitment of other immune cells that conduce to neuronal damage. However, when the injurious stimuli are controlled, the inflammatory response is diminished by cytokines with anti-inflammatory activity such as transforming growth factor (TGF-β) and IL-10 by microglia. Therefore, the imbalance of microglial activation could exacerbate the pro-inflammatory response, leading to neuronal degeneration and cell death in AD and PD, and neuropathic and inflammatory pain (Suter et al., 2007; Ji et al., 2013; Beggs and Salter, 2016; Carniglia et al., 2017).

Some kinases have a described role in pain. It was reported that extracellular signal-regulated kinases 1/2 and 5 (ERK1/2 and ERK5) are expressed in microglia, and their phosphorylation is induced during neuropathic pain (Tatsumi et al., 2015; Carniglia et al., 2017). Furthermore, it was observed that neuropathic pain induced by nerve injury, promoted the phosphorylation of p38 mitogen-activated protein kinase (MAPK) in spinal microglia. p38 MAPK is activated by multiple microglial receptors, inflammatory cytokines, membrane depolarization, and Ca^2+^ influx. This pathway regulates pro-inflammatory signaling networks as well as the production of diverse inflammatory molecules associated with pain facilitation, including the cytokines TNF-α and IL-1β (Ji and Suter, 2007; Lee and Kim, 2017). Moreover, it was confirmed that the inhibition of p38 MAPK decreases the release of pro-inflammatory cytokines, inducing relieve of mechanical allodynia in diverse models of neuropathic and inflammatory pain (Jin et al., 2003; Lee and Kim, 2017; Inoue and Tsuda, 2018). Interestingly, in addition to their role in pain, the p38 MAPK pathway has also been involved in the cellular mechanisms that regulate neurodegeneration (Lee and Kim, 2017; Kheiri et al., 2019). Activated p38 MAPK was observed in peripheral blood leukocytes and neuronal cells, as well as in postmortem brain from patients with AD (Sun et al., 2003; Kheiri et al., 2019).

p38 MAPK role in AD has been associated with both Tau protein and Aβ peptide, which are essential players in AD pathologies. For instance, Aβ peptide promotes the activation of p38 MAPK, which in turn, phosphorylates Tau protein in neuronal cells (Lee and Kim, 2017). In this vein, it has been described that Aβ peptides suppress nociception and inflammatory pain in APP overexpressing CRND8 transgenic mice (Shukla et al., 2013); this finding is in accordance with the finding that mice treated with a single intracerebroventricular injection of Aβ fragment (1–40) (400 pmol/mice) displayed increased pain tolerance (Pamplona et al., 2010). However, pain sensitivity could be altered in a more complex form since i.c.v. Aβ treated mice also display anxiogenic-like and depressive-like states, which are related to alterations in cognitive/emotional components of pain processing (Pamplona et al., 2010). Also, it has been described that Tau depletion, *in-vivo* studies, negatively affects the main systems conveying nociceptive information to the CNS (Sotiropoulos et al., 2014). Tau-null (Tau−/−) mice display reduced C-fiber density and Aδ-fiber hypomyelination followed by diminished conduction properties sciatic nerves and decreased nociception but increased excitability of second-order spinal cord nociceptive neurons, resulting in heightened pain-like behaviors (Sotiropoulos et al., 2014; Lopes et al., 2016). These findings suggest that APP and Aβ peptides and Tau protein could affect in a complex way pain perception in AD patients.

Several reports also suggest that p38 MAPK is also involved in PD. It is proposed that oxidative stress in dopaminergic neurons prompted the activation of the p38 MAPK and c-Jun N-terminal kinase (JNK) signaling pathways that have linked to neuronal apoptosis in several models of PD (Oh et al., 2011; Sabens Liedhegner et al., 2011; Bohush et al., 2018). p38-MAPK activation has also been reported to contribute to mitophagy, a fundamental mechanism underlying α-synuclein accumulation associated with PD (Cheng et al., 2018).

TRP channels function has been related to p38 MAPK pathway activity. It has been reported that phosphorylated p38 MAPK stimulated by noxious cold colocalized in neurons that express TRPA1 channels (Mizushima et al., 2006). Additionally, stimulation of microglia with lipopolysaccharide and interferon γ (LPS/IFN γ) promoted the activation of TRPM2 channels and Ca^2+^ dependent signaling pathways, and the increase in p38 MAPK signaling (Miyake et al., 2014). Interestingly, the use of TRPM2 inhibitors inhibited the extracellular Ca^2+^ influx, affecting the activation of the p38 MAPK pathway. Similar results have been observed in TRPM2-KO microglia, where NO release was attenuated (Haraguchi et al., 2012). It is suggested that TRPM2 recruits the p38 MAPK pathways for NO production induced by LPS/INFγ. Furthermore, phosphorylation of p38 MAPK was abolished in TRPM2-knockout microglia, indicating that this process is selectively dependent on TRPM2 signaling. Similarly, lisophosphatidylcholine (LPC), an endogenous inflammatory phospholipid that induces TRPM2 translocation to the plasma membrane, also promotes Ca^2+^ influx and microglia activation. It has been demonstrated that LPC increases phosphorylation of p38 MAPK in microglia, which was eliminated in TRPM2-KO. From these results, it is feasible to propose TRPM2 channels as potential therapeutic targets to inhibit excessive microglial activation, neuroinflammation, and, therefore, pain through modulation of p38 MAPK phosphorylation (Miyake et al., 2014; Jeong et al., 2017; Shirakawa and Kaneko, 2018).

Considering that the p38 MAPK pathway is a central player in neurodegeneration and pain, several recent studies have been focused in search of p38 MAPK activity modulators, and some molecules have shown anti-inflammatory activity (Jeong et al., 2017; Kheiri et al., 2019). However, cross-reactivity with other kinases and the appearance of cardiovascular, psychiatric, and hepatic side effects have halted the use of these molecules, suggesting that it is necessary to study further the mechanism by which p38 MAPK could be modulated to avoid the adverse side effects observed (Ji and Suter, 2007; Kheiri et al., 2019).

In addition to the regulation of p38 MAPK phosphorylation in microglia, TRP channels also play a role in the generation of peripheral pain through oxidative stress. Oxidative stress-mediated by lipid peroxidation has been observed in both neurological and peripheral pain. It has been proposed that selenium could act as neuroprotector through a mechanism that involves TRP channels inhibition, which in turn, induces modulation of ROS overproduction and Ca^2+^ influx (Nazıroğlu et al., 2020). Selenium is an inhibitor of TRPM2 channels, which reduces oxidative stress in the cytosol (Zeng et al., 2012). Besides TRPM2, selenium also acts as TRPA1 and TRPV1 inhibitor, suggesting that selenium could be used as a modulator of neuropathic pain through TRP channel modulation (Nazıroğlu et al., 2020).

Despite high rates of painful comorbidities, lower use of analgesics among individuals with dementia has been reported (Van Kooten et al., 2016). Detriment in pain management seems to occur in part due to challenging pain assessment in patients with compromised cognition and impaired communication skills, as well as barriers to analgesics (Shen et al., 2018).

Currently, several families of agents have been of clinical utility to treat pain. The most common analgesic drug prescribed for mild to moderate pain is paracetamol (also known as acetaminophen); however, for peripheral or central neuropathic pain, this analgesic drug has poor effectiveness. Opioids, anticonvulsants, nonsteroidal anti-inflammatory drugs (NSAIDs), topical medications, and more recently, third-generation antidepressants have been used to treat pain related to nerve injury (Lynch and Watson, 2006; Yaksh et al., 2015). However, important drugs safety and side effects limit their use; this is particularly important in the case of opioids, which are the most effective pain killers but have high potential to induce addiction and may cause sedation and respiratory depression (Moran and Szallasi, 2018). Clinical daily work shows that the use of painkillers, opioids, antidepressants, or anticonvulsive drugs are often not sufficient to treat pain in neurodegenerative diseases, so it has been suggested that in selected individuals, refractory to conventional treatment of pain, cannabinoid management could be attempted (Jost and Buhmann, 2019). It has been recently shown that cannabinoids provide promising multitarget approach for the treatment of pain and neurodegeneration since they regulate the activity of TRP channels, which are considered non-cannonical endocannabionoid receptors. In this vein, it has been shown that cannabidiol, cannabinol, cannabigerol, or cannabidiolic acid binds TRPs, including TRPV1–4, TRPA1, and TRPM8 (Shirakawa and Kaneko, 2018; Muller et al., 2019; Starkus et al., 2019).

Since TRPs are involved in the progression of neurodegenerative diseases and have a role in pain, they are remarkable potential targets for the treatment of both pain and neurodegenerations (Zündorf and Reiser, 2011; Naziroğlu, 2012; Maiese, 2017; Echeverry et al., 2016; Belrose and Jackson, 2018). Recent evidence regarding the involvement of TRP channels in several diseases has led to the identification of TRP channels as potential drug targets to manage pain. For instance, capsaicin, an agonist of TRPV1, has been used in clinical trials to control neuropathic pain conditions (Kiani et al., 2015; Derry et al., 2017), however, its use would be limited by two major adverse effects of TRPV1 channel agonists/antagonists: (a) dysregulation of body temperature, and (b) long-lasting compromise of temperature sensation leading to burning injuries. Agents targeting TRPM8, TRPV2, TRPV3, TRPV4, and TRPA1 have also been tested with mixed results. Interestingly, in animal models, TRPA1 deletion or inhibition reduces pain associated with inflammation, as well as inflammation *per se* (Moilanen et al., 2015, 2016; Horváth et al., 2016). A role for TRPA1 channels in neurogenic inflammation has been suggested (Moran and Szallasi, 2018); indeed, a Phase 2 clinical trial has have reported that the Glenmark’s GRC 17536 TRPA1 channel antagonist significantly reduce pain scores in a pre-specified subset of patients with painful diabetic neuropathy and intact sensory responses without notable side effects (Moran and Szallasi, 2018). Recently, TRPM2 inhibitors have been proposed as a potential candidate to treat neurodegeneration and pain, and several novel molecules targeting TRPM2 (8Br-ADPR, 8-Ph-2’-deoxy-ADPR and novel ADPR analogs capable of selectively inhibiting TRPM2) appear as potential candidates to develop novel therapeutic agents (Belrose and Jackson, 2018). Notably, a cell-permeable peptide tat-M2NX that inhibits TRPM2 provides protection from ischemic stroke in adult mice decreases infarct volume with a clinically relevant therapeutic window (when provided either prior to the infarct or 3 h following the insult) (Shimizu et al., 2016).

Since TRP channels are involved in numerous physiological processes, attention should be paid to potential side effects of drugs able to block TRP channels their function. Concerns predominantly relate to the roles of TRP channels in temperature sensation and regulation, immune function, and insulin release (Belrose and Jackson, 2018). Ultimately, assessment of the risk-benefit profile of TRPs as therapeutic targets will require the development of specific compounds with favorable pharmacokinetic and pharmacodynamic properties and identification of specific patient populations that would benefit the most (Belrose and Jackson, 2018). In this vein, it would be worth testing selective drugs targeting TRPs to manage neurodegeneration and treat associated symptoms such as pain and cognitive/motor dysfunction. The evidence suggests that the effectiveness of pharmacological agents regulating TRP channel activity to treat neuropathological processes and pain deserves further research. Evaluation of the risks and benefits of TRPs’ use as therapeutic targets will need the development of compounds with favorable pharmacological properties and identification of specific patient populations that would benefit the most. In this regard, it would be worthy of testing selective drugs targeting TRPs to manage neurodegeneration and treat associated symptoms, such as pain and cognitive/motor dysfunctions. Furthermore, given that TRPs are involved in the progression of neurodegenerative diseases and have a role in pain, it is feasible to propose that these channels could act as central players that connect both processes, making TRP channels potential targets to treat pain in NDDs patients (Zündorf and Reiser, 2011; Naziroğlu, 2012; Echeverry et al., 2016; Maiese, 2017). Direct evidence describing the role of TRPs on pain related to NDDs development is still required, however, indirect evidence suggests that this subject deserves further research and supposes and interesting field of research.

## Author Contributions

MD, VV-L, ZC, SA, J-JS, and YT wrote the manuscript. All authors contributed to the article and approved the submitted version.

## Conflict of Interest

The authors declare that the research was conducted in the absence of any commercial or financial relationships that could be construed as a potential conflict of interest.
